# Neoadjuvant irradiation of retroperitoneal soft tissue sarcoma with ions (Retro-Ion): study protocol for a randomized phase II pilot trial

**DOI:** 10.1186/s13063-021-05069-z

**Published:** 2021-02-12

**Authors:** K. Seidensaal, M. Kieser, A. Hommertgen, C. Jaekel, S. B. Harrabi, K. Herfarth, G. Mechtesheimer, B. Lehner, M. Schneider, H. Nienhueser, S. Fröhling, G. Egerer, J. Debus, M. Uhl

**Affiliations:** 1 Department of Radiation Oncology, Heidelberg University Hospital, Heidelberg, Germany; 2 Heidelberg Institute of Radiation Oncology (HIRO), Heidelberg, Germany; 3 National Center for Tumor diseases (NCT), Heidelberg, Germany; 4 Institute for Medical Biometry and Informatics, University of Heidelberg, Heidelberg, Germany; 5 Clinical Cooperation Unit Radiation Oncology, German Cancer Research Center (DKFZ), Heidelberg, Germany; 6 Heidelberg Ion-Beam Therapy Center (HIT), Department of Radiation Oncology, Heidelberg University Hospital, Heidelberg, Germany; 7 Institute of Pathology, University of Heidelberg, Heidelberg, Germany; 8 Center for Orthopedics, Trauma Surgery and Paraplegiology, University of Heidelberg, Heidelberg, Germany; 9 Department of General, Visceral and Transplantation Surgery, University Hospital Heidelberg, Heidelberg, Germany; 10 Department of Translational Medical Oncology, National Center for Tumor Diseases Heidelberg and German Cancer Research Center, Heidelberg, Germany; 11 Department of Hematology, Oncology and Rheumatology, Heidelberg University, Heidelberg, Germany; 12 German Cancer Consortium (DKTK), partner site Heidelberg, Heidelberg, Germany

**Keywords:** Retroperitoneal soft tissue sarcoma, Carbon ion therapy, Proton therapy, Irradiation, Randomized trial, Hypofractionation, Heavy ion therapy

## Abstract

**Background:**

Following surgery for soft tissue sarcoma of the retroperitoneum, the predominant pattern of failure is local recurrence, which remains the main cause of death. Radiotherapy is utilized to reduce recurrence rates but the efficacy of this strategy has not been definitely established. As treatment tolerability is more favorable with preoperative radiotherapy, normofractionated neoadjuvant treatment is the current approach. The final results of the prospective, randomized STRASS (EORTC 62092) trial, which compared the efficacy of this combined treatment to that of surgery alone, are still awaited; preliminary results presented at the 2019 ASCO Annual Meeting indicated that combined treatment is associated with better local control in patients with liposarcoma (74.5% of the cohort, 11% benefit in abdominal progression free survival after 3 years, *p* = 0.049). Particles allow better sparing of surrounding tissues at risk, e.g., bowel epithelium, and carbon ions additionally offer biologic advantages and are preferred in slow growing tumors. Furthermore, hypofractionation allows for a significantly shorter treatment interval with a lower risk of progression during radiotherapy.

**Methods and design:**

We present a prospective, randomized, monocentric phase II trial. Patients with resectable or marginally resectable, histologically confirmed soft tissue sarcoma of the retroperitoneum will be randomized between neoadjuvant proton or neoadjuvant carbon ion radiotherapy in active scanning beam application technique (39 Gy [relative biological effectiveness, RBE] in 13 fractions [5–6 fractions per week] in each arm). The primary objective is the safety and feasibility based on the proportion of grade 3–5 toxicity (CTCAE, version 5.0) in the first 12 months after surgery or discontinuation of treatment for any reason related to the treatment. Local control, local progression-free survival, disease-free survival, overall survival, and quality of life are the secondary endpoints of the study.

**Discussion:**

The aim of this study is to confirm that hypofractionated, accelerated preoperative radiotherapy is safe and feasible. The rationale for the use of particle therapy is the potential for reduced toxicity. The data will lay the groundwork for a randomized phase III trial comparing hypofractionated proton and carbon ion irradiation with regard to local control.

**Trial registration:**

ClinicalTrials.gov NCT04219202. Retrospectively registered on January 6, 2020

## Background

Retroperitoneal sarcomas constitute approximately 15% of all soft tissue sarcomas [1, 2]. As the early stages can remain without specific signs or symptoms for a long time, many patients are often diagnosed with large tumors. The main histology is lipo- or leiomyosarcoma and about 60% are high-grade tumors [3]. The primary treatment of patients with retroperitoneal sarcomas is surgery; however, the close proximity to intraabdominal organs hinders resections with wide margins in many cases and about 45% of tumors are resected incompletely (R1/R2) [4]. The risk of local failure is generally high especially when a complete resection cannot be achieved. The general policy for local recurrence is re-operation. Retroperitoneal sarcomas show a lower risk for distant metastases; local control is crucial as the primary cause of death in these patients is local disease recurrence [4–6]. In contrast to extremity sarcomas, most patients die without distant metastases. Preoperative radiotherapy seems to be able to reduce the risk of local recurrence based on data from small sample sized studies and retrospective analyses [1]; at many centers, external beam radiotherapy is combined with an intraoperative radiotherapy [7]. The final publication from the only prospective trial comparing surgery with or without neoadjuvant conventional photon radiotherapy is still awaited. Until now, the role of radiotherapy is substantiated on the role in extremity sarcoma. The toxicity of a preoperative treatment is significantly lower compared to a postoperative treatment; however, the potential for toxicity is significant based on the typically large tumor volumes and precise radiation techniques are required. The special physical and biological properties of protons and carbon ions lead to a superior dose distribution [8, 9]. This allows a better sparing of the surrounding organs at risk, with chances to reduce treatment-related side effects and even potential for dose escalation. Considering the typically large target volumes, reduced dose to the surrounding bowel, kidney, and the hematopoietic tissue in the bone marrow is particularly desirable. Primary phase I data for neoadjuvant proton radiotherapy show promising results with mild toxicity [10]. Additional carbon ions have a higher biological effectiveness and are promising in the treatment of sarcoma and chordoma. The standard neoajuvant normofractionated radiotherapy consists of 25 fractions with 2-Gy single dose over 5 weeks; the special properties of particles allow hypofractionation resulting in a reduced treatment total time of 3 weeks.

Herein we present a single-institution, prospective, randomized trial for a neoadjuvant hypofractionated carbon ion or proton radiotherapy of retroperitoneal sarcoma.

## Methods and design

### Primary objective

The primary objective of this trial is the evaluation of safety and feasibility of neoadjuvant hypofractionated irradiation in patients with retroperitoneal sarcoma using ions (protons or carbon ions) in raster scan technique. The primary endpoint is defined as the proportion of patients treated without grade 3–5 toxicity (CTCAE) up to 12 months after treatment and without discontinuation of the treatment for any reason.

### Secondary objectives

Assessment of local control (LC) and local progression-free survival (LPFS) and disease-free survival (DFS). Further objectives are overall survival (OS) from the start of treatment until death or censoring and quality of life (QoL) using the EORTC-QLQ30 questionnaire.

### Study design

**Fig. 1:**
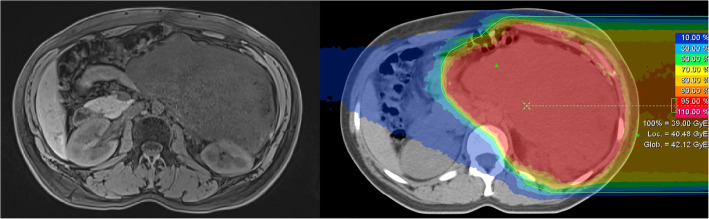
MRT and dose distribution of the carbon ion plan of a 33-year-old patient with first diagnosed retroperitoneal liposarcoma

The study is a parallel-group prospective clinical phase II trial of patients with retroperitoneal sarcoma, randomized to one of the two treatment arms (arm A: proton therapy, arm B: carbon ion therapy). A total dose of 39 Gy (RBE) in 13 fractions to the PTV (see target definition below) will be given in both arms. The accrual period of this trial will take approx. 4 years with a follow-up time of 12 months for each patient. Patients matching the eligibility criteria and willing to participate with written informed consent are registered at Heidelberg University Hospital (Fig. 1).

### Inclusion criteria

i) Histological confirmation of non-metastatic retroperitoneal soft tissue sarcoma, which is resectable or marginally resectable
ii) Karnofsky performance status ≥ 70%
iii) Patients age above or equal 18
iv) Written informed consent

### Exclusion criteria

i) Distant and/or lymph node metastases
ii) Desmoid tumors, gastrointestinal stromal tumor (GIST), peritoneal sarcomatosis
iii) Metal implants at the level of the tumor that could influence treatment planning
iv) Inability of the patient to lie quietly for at least 20 min (e.g., due to pain)
v) Prior radiotherapy to the abdominopelvic cavity
vi) Simultaneous participation in another trial that could influence the results of the study
vii) Active medical implants without treatment approval at the time of ion irradiation (e.g., cardiac pacemaker, defibrillator)
viii) Pregnancy

### Treatment planning and target volume delineation

Examinations for treatment planning consist of a CT scan (3-mm slice thickness) in treatment position and a MRI for 3D image correlation. The delineation of the retroperitoneal soft tissue sarcoma requires a T1-weighted post-gadolinium sequence.

Gross tumor volume (GTV) includes the gross tumor based on CT and MRI imaging. The clinical target volume (CTV) is defined as GTV plus surrounding areas at risk for containing microscopic disease. The CTV includes the GTV with a margin of 2 cm. The CTV margins may be smaller if the GTV is adjacent to the critical normal organs like the small bowel. The planning target volume (PTV) includes the CTV with an additional margin of 5 mm in the anterior–posterior direction and 7 mm in the lateral direction to compensate set-up variability. The overlap of PTV and the rectum or intestine is defined as PTV-rectum or PTV-intestine.

### Proton/carbon ion therapy

Treatment will be administered on an outpatient basis at the Heidelberg Ion-Beam Therapy Center (HIT) exclusively in active raster scanning technique [11, 12]. Apart from the standard supportive treatment, no other treatment will be administered in parallel to radiotherapy. Treatment planning is realized using a treatment planning system (TPS Siemens) that enables conventional and biological optimization. After inverse planning, proton and carbon ion treatment is given in active beam application (raster scanning method).

### Dose prescription and constraints of critical organs at risk

Ninety-five percent of the PTV should obtain the dose of 39 Gy (RBE) in 13 fractions (5–6 fractions per week). The rectum, bladder, intestine, and kidney will be defined as organs at risk depending on the exact tumor localization. It is assumed that the *α*/*β* value of soft tissue sarcoma is < 10; a consistent value cannot be found in literature. The equivalent photon dose in 2 Gy fractions (EQD2) is in the range of 48.8–42.2 Gy for a *α*/*β* = 2–10. The maximal dose for PTV-intestine and PTV-rectum is 39 Gy (RBE) (100% isodose). The EQD2 is 45.5 Gy for the intestine (*α*/*β* = 4.0 Gy) and 45.6 Gy (*α*/*β* = 3.9 Gy). This dose will be allowed to a small volume only and not circumferentially. The maximum dose for the myelon and cauda equina is 37.0 Gy (RBE) (95% isodose), and the EQD2 is 44.9 Gy. If no nephrectomy is planned, two-thirds of the kidney volume should receive less than 26.5 Gy (RBE) (68% isodose) and one-third should receive less than 33.5 Gy (86% isodose). The Quantitative Analyses of Normal Tissue Effects in the Clinic (QUANTEC) criteria for the rectum, bladder, and the further bowel will be followed (V50 < 50%, V60 < 35%, V65 < 25%, V70 < 20%, and V75 < 15%).

### Toxicity, safety, and quality of life

Toxicity will be evaluated according to International Common Terminology Criteria for Adverse Events (CTCAE) version 5.0 for toxicity (case report forms) and adverse event reporting including the results of clinical examinations as well as imaging studies (MRI or CT). At enrollment, each patient will pass a baseline clinical examination. During treatment, the patient is monitored continuously and acute adverse events will be documented weekly and at the end of treatment. Three weeks after the end of radiotherapy, the patient will receive an MRI, and the surgery is planned 4–6 weeks after RT. The further follow-up presentations are 6 weeks and 3, 6, 9, and 12 months after the tumor resection and include all imaging studies (CT/MRI) (Table 1). The criteria for feasibility and safety are fulfilled, if no grade 3 or higher toxicity has occurred from the start of irradiation until 12 months after surgery, and if the therapy was not canceled due to any reason (unless clearly not treatment related).

**Table 1:** Time flow of procedures and examinations

	Study inclusion	Prior to RT	During RT	At the end of RT	3 weeks after RT	OP: 4–6 weeks after RT	6 weeks after RT	3 months after OP	6 months after OP	9 months after OP	12 months after OP
Inclusion and exclusion criteria	x										
Informed consent	x										
Medical history	x		x	x	x		x	x	x	x	x
Evaluation of symptoms and toxicity	x		x	x	x		x	x	x	x	x
Radiotherapy			x								
Resection						x					
MRI abdomen/pelvis		x			x			x	x	x	x
CT thorax		x						x	x	x	x
Quality of life (QLQ-C30)		x			x			x			x

Quality of life (QoL) will be assessed by the means of the EORTC QLQ-C30 questionnaires. Those will be filled out by the patient before treatment, 3 weeks after the end of RT, 6 months after surgery, and at the end of the observation interval of 12 months. The evaluation will be carried out after the complete acquisition of data. Changes in QoL will be determined comparing the results before and after radiotherapy as well as the difference before and after surgery. QoL will be compared between the two arms of the study after the end of radiation and at the end of the observation interval.

### Secondary endpoints

The effectiveness of treatment is examined through MRI and CT follow-ups. Local control rate is defined as freedom from local progression, and local progression-free survival is defined as survival probability from the beginning of radiotherapy until local progression. Disease-free survival is defined as the disease-free time from the beginning of radiotherapy until local or distant progression. The overall survival as a secondary endpoint will be defined as the time from the beginning of radiotherapy until the date of death due to any reason, until the end of the observation period or until the date of the last presentation in case of loss to follow-up (censoring).

### Statistical consideration and analysis

This study is performed to deliver basic data for the neoadjuvant hypofractionated carbon ion or proton radiation treatment of patients with retroperitoneal soft tissue sarcoma by providing safety and toxicity data for the here presented treatment regimen. The standard treatment is a neoadjuvant normofractionated photon radiotherapy and is considered as the historical cohort which served for the planning of this pilot study including the framework of the safety and feasibility considerations. The randomization will be performed as block randomization at a ratio of 1:1 aiming at equal group sizes by using a web-based randomization tool (randomizer.at).

The primary endpoint is assessed by calculating the two-sided 95% Agresti–Coull interval [13] for each treatment group. The considerations on the choice of the sample size are based on this analysis approach. It is assumed that the proportion of patients treated without grade 3–5 toxicity (CTCAE) up to 12 months after treatment and/or without discontinuation of the treatment for any reason is 0.90. The lower boundary of the two-sided 95% confidence interval should then not fall below 0.75. To achieve this goal under the above assumption, *n* = 32 patients per arm are required.

The intention-to-treat population (ITT) is the primary population for the primary and secondary endpoints and consists of all patients who were included in the study and were treated at least once. The per-protocol population consists of all patients who obtained the allocated treatment as planned and whose documentation is complete. The safety population includes those patients who started treatment (at least 1 day); this is the primary cohort for the analysis of all safety variables.

Due to the pilot character of the trial, all analyses are performed descriptively and the results are to be interpreted accordingly. For the primary endpoint, two-sided 95% Agresti–Coull interval is calculated for each treatment group. If missing values occur, multiple imputation based on the “fully conditional specification” method [14] is applied, where treatment group allocation is taken into account in the imputation procedure. Additionally, a complete-case analysis and an analysis according to the ICA-r method are performed [15]. Furthermore, a descriptive comparison of the treatment groups is conducted by application of the chi-square test. Analysis of the secondary time-to-event endpoints LPFS, DFS, and OS is performed by applying the Kaplan–Meier method, where patients without event are censored. The treatment groups are descriptively compared using the logrank test. The secondary endpoint quality of life measured by the EORTC-QLQ30 questionnaire is assessed by presentation of mean, standard deviation, median, minimum, and maximum as well as application of a descriptive two-sample *t* test. For the secondary endpoint local control, absolute and relative frequencies are calculated for each treatment group together with two-sided 95% Agresti–Coull interval and a descriptive chi-square test will be applied for treatment group comparison. Adverse and serious adverse events will be listed, and absolute and relative frequencies will be calculated.

Statistical analysis will be performed by the statistical software SAS v9.4 (SAS Institute, Cary, NC).

The descriptive significance level is throughout 5%, two-sided; all *p* values are to be interpreted as descriptive; and there is no adjustment for multiplicity.

There will be no planned interim analysis. The results of this study will facilitate a subsequent phase III trial.

### Data Safety Monitoring Board

A Data and Safety Monitoring Board (DSMB) consisting of independent experts in the field of radiation oncology will monitor the recruitment, the reported (serious) adverse events, and the quality of data. The goal of the DSMB is to ensure that the study is executed according to current standards of good clinical praxis with focus on the safety interest of the patients. The DSMB will provide the principal investigator (PI) with recommendations regarding trial modification, continuation, or premature termination.

### Regular study end

The estimated period of patient accrual is 4 years. The regular end of the treatment period for each patient is 2 to 3 weeks after initiation of radiation therapy (13 fractions, 5–6 fractions per week). The regular end of study participation for each patient is after a follow-up period of 12 months after surgery.

### Premature study termination

Reasons for premature termination of the entire study are:

i) Unacceptable risks or toxicities (assessment by DSMB)
ii) Occurrence of one toxicity of grade 5 or two consecutive grade 4 toxicities or five consecutive grade 3 toxicities unequivocally associated with the study therapy. The decision whether a toxicity equal or higher than grade 3 is associated with the study treatment is made by the DSMB
iii) Furthermore, new scientific findings during the period of study incompatible with the study treatment

Individual reasons for premature study termination are serious events during the radiotherapy (grade 4 or 5) or the case that a patient withdraws consent.

### Collection and management of trial-related data

All patient-related data are collected pseudonymously and allocated to individual patient numbers. The study data are gathered in form of case report forms. According to the German GCP-Regulation, all important trial documents will be archived for at least 10 years after the end of the trial. According to the German Radiation Protection Regulation (StrlSchV), the documentation of written informed consent including the patients’ consent for trial and the documentation of irradiation will be archived for at least 30 years. The Study Center at the Department of Radiation Oncology will be responsible for archiving all relevant data. The patients will be informed that the disease-associated data will be saved pseudonymously for scientific evaluation of the study results. The patient can request access to the saved data. Access to original files can be only granted by the principal investigator or commissioned by the state authorities. If the patient withdraws informed consent, the so far acquired data material will be destroyed if the patient does not agree with further use and analysis.

The trial is conducted in accordance with the Declaration of Helsinki (2008 version of the Declaration of Helsinki, adopted at the 59th WMA General Assembly, Seoul, October 2008) as well as with the guidelines of Good Clinical Practice (ICH-GCP: International Conference on Harmonization - Good Clinical Practice; May 1, 1996) in their current versions.

## Discussion

With the here presented study protocol, we hypothesize that a neoadjuvant hypofractionated particle radiotherapy with either protons or carbon ions is safe and feasible.

To date, the role of radiotherapy as an adjunct to surgery in the treatment of retroperitoneal sarcoma is controversial and several retrospective studies in favor or against radiotherapy have been published [16–20]. This discrepancy in comparison to extremity soft tissue sarcomas might lie in biologic differences and tumor-specific characteristic. However, in comparison to extremity sarcomas, radiotherapy of retroperitoneal sarcomas is technically more complex, steep dose fall offs to the adjacent organs at risk (small intestine, kidney, spinal cord) are required, and with conventional treatment techniques target volume coverage can be compromised in order to comply to the risk organ constrains. In the postoperative setting, which is included in many retrospective cohorts, these challenges are even more prominent and aggravated by difficulties in defining the postoperative tumor bed as a surrogate for microscopic residual disease and the required doses of 60–66 Gy are not reached in many cases. Despite the high grade of conformality of the modern photon techniques, toxicity equal or higher than grade 3 is seen in approximately 15% of patients before surgery and 33% of patients after surgery [21]. The benefit of particles lies in the steeper dose gradients, which improve the chances of homogeneous target volume coverage and reduction of dose constraints considered relevant for, e.g., diarrhea as V40Gy < 124 cm^3^ [22], V15Gy < 275 cm^3^ [23]. One previous plan comparison investigated the differences in neoadjuvant proton and photon radiotherapy. Protons showed reduced integral dose to the surrounding organs at risk, which might result in less gastrointestinal and genitourinary toxicity [24]. However, this superior level of precision has special demands on treatment implementation. The dose distribution of particles is more sensitive to deviations from the planned anatomy. Small changes as positioning inaccuracies, variable fillings of hollow organs, or increased muscle tension can change the range and the position of dose in the patient. Neoadjuvant intensity-modulated proton therapy has been investigated in a phase I pilot study (*n* = 11), which showed only mild toxicity and no unexpected perioperative morbidity. In this study, a dose escalation was performed to the high-risk PTV from 60.2 Gy (RBE) up to 63 Gy (RBE) in 28 fractions as a simultaneous integrated boost. The average risk PTV received the fixed dose of 50.4 Gy (RBE) [10]. Thus, further investigation of the dosimetric advantages of particles and their translation into clinical benefits is urgently required.

With the advances in radiotherapy treatment techniques, hypofractionation is becoming a growing trend in radiotherapy of sarcoma [25, 26]. The precise *α*/*β* ratio of retroperitoneal soft tissue sarcomas has yet to be established but nonetheless a high fractionation sensitivity is currently assumed (*α*/*β* < 4–5), and thus, hypofractionation seems to be beneficial in regard to the radiobiological response of sarcoma cells [27]. The technical advantages of particles are utilized in order to compensate for the potential aggravation of side effects by the implementation of hypofractionation in this protocol. Additionally, in this trial, the overall radiotherapy time is reduced by half through hypofractionation. This might not only improve the patient’s quality of life but also reduce the risk of progression in the timeframe prior to surgery as has been observed in the STRASS trial (see below). Potential risk lies additionally in the uncertainties in the definition of the equivalent total dose to the standard normofractionated schedule of 50 Gy in 25 fractions.

Carbon ion therapy has been investigated in the definitive treatment of inoperable retroperitoneal sarcoma of various histologies including malignant fibrous histiocytoma, liposarcoma, malignant peripheral nerve sheath tumor, and Ewing sarcoma/primitive neuroectodermal tumor with local control rates of 75% and 50% after 2 and 5 years (median follow-up 36 months) and no higher-grade toxicity above grade 2. The applied total and single dose was higher, 52.8 to 73.6 GyE in fixed 16 fractions [28]. However, the RBE model is differing between the Japanese institutions and the German facilities and thus comparison regarding toxicity and dose is limited. Further investigation especially of the clinical differences of protons and carbon ions is urgently necessary.

One of the biggest previously published study cohorts on radiotherapy of retroperitoneal soft tissue sarcoma is a case–control, propensity score-matched analysis of a nationwide database of 9068 patients which supports the role of RT in the treatment of retroperitoneal soft tissue sarcoma. The trial compared patients who received preoperative radiotherapy to patients who received no radiotherapy and patients who received postoperative radiotherapy compared to no radiotherapy. Both radiotherapy groups showed a survival benefit. The 5-year survival was 62% in the preoperative therapy group and 54% in the no radiotherapy group (HR 0.70, 95% CI 0.59–0.82; *p* < 0.0001). The median survival was 110 months in the preoperative RT group and 66 months in the no RT group. In the postoperative radiotherapy group, the median survival was 60% for patients receiving postoperative RT and 52% for patients receiving no RT. The median survival was 89 months for the postoperative RT group and 64 months for those receiving no RT (HR 0.78, 95% CI 0.71–0.85; *p* < 0.0001) [1].

The randomized phase III STRASS trial is the first randomized trial assessing neoadjuvant RT for 50.4 Gy in 28 fractions for retroperitoneal sarcoma. In total, 266 patients were enrolled and randomly assigned to surgery only or preoperative RT and surgery. In the RT group, 95% of patients received IMRT. In October 2020, the preliminary data with a median follow-up of 43 months were reported in *Lancet Oncology*. The primary endpoint was an increase of the abdominal recurrence-free survival (ARFS) by 20% at 5 years, which was not reached so far. However, in the group of liposarcoma (74.5%), the 3-year ARFS was 71.6% in the surgery and RT group and 60.4% in the surgery group alone [29]. The composite primary endpoint which is not validated consisted of local or distant progression during or after RT, inoperability of tumor or patient defined as ASA score of 3, R2 resection, peritoneal metastases, or local recurrence after resection. There were twice as many local relapses in the surgery only compared to the radiotherapy and surgery group (*N* = 39 vs *N* = 17); thus, the sub-endpoint local recurrence appears clearly in favor of RT. The sub-endpoint progression during radiotherapy assessed on computed tomography 2 weeks after the end of RT was the factor that led to no difference in ARFS. Still, 14 of 16 patients declared as progressed locally received surgery. Furthermore, there were more patients declared inoperable based on the ASA scoring system in the surgery plus radiotherapy group who then received complete resection [30]. Interesting primary endpoints would have been local control alone and survival. There may be some benefit of RT in specific subtypes as liposarcoma and not in others as leiomyosarcoma; however, the trial was not powered to evaluate specific subtypes. Further follow-up might provide additional insight in the future.

## Conclusion

Up to date, the role of neoadjuvant radiotherapy for soft tissue sarcoma is not clearly defined and requires further investigation. The aim of this study is to confirm that a hypofractionated, accelerated preoperative radiotherapy is safe and feasible. The rational of the use of particle therapy is the potential of reduced toxicity.

Investigating this approach is promising as it bears the chances of superior local control compared to surgery alone and lower toxicity compared to photon radiotherapy, at the same time reducing the total treatment time. The data will facilitate a further randomized phase III trial, comparing hypofractionated proton and carbon ion irradiation in regard to local control.

## Trial status

Protocol version 2.2, September 1, 2018; the recruitment began on April 30, 2019; the approximate date of recruitment completion is May 2023.

## Data Availability

Not applicable

## Abbreviations

ASA: American Society of Anesthesiologists
ARFS: Abdominal recurrence-free survival
ASCO: American Society of Clinical Oncology
CTCAE: Common Terminology Criteria for Adverse Events
CTV: Clinical target volume
DFS: Disease-free survival
DSMB: Data and Safety Monitoring Board
GTV: Gross tumor volume
HIT: Heidelberg Ion-Beam Therapy Center
ICA-r: Independent component analysis with reference
IMRT: Intensity-modulated radiotherapy
ITT: Intention to treat
LC: Local control
LPFS: Local progression-free survival
OS: Overall survival
PI: Principal investigator
PFS: Progression-free survival
PTV: Planning target volume
QoL: Quality of life
RBE: Relative biological effectiveness
RT: Radiotherapy
